# Verbal and Social Autopsy of Adult Deaths and Adult Care-Seeking Pattern in Mozambique, 2019–2020

**DOI:** 10.4269/ajtmh.22-0548

**Published:** 2023-04-10

**Authors:** Md Hafizur Rahman, Ivalda Macicame, Emily Wilson, Sheila Nhachungue, Agbessi Amouzou

**Affiliations:** 1 Johns Hopkins University Bloomberg School of Public Health, Baltimore, Maryland;; 2 Instituto Nacional de Saúde, Maputo, Mozambique

## Abstract

In sub-Saharan Africa, recent data about causes of adult death and care-seeking during illnesses are limited. This analysis examines adult deaths using verbal and social autopsy data from a nationally and provincially representative sample registration system in Mozambique. Causes of death among those 18 years and older were assigned using the InSilicoVA algorithm, and underlying social causes were examined using the pathway to survival model. Care-seeking was analyzed in different groups to determine if care was sought from formal providers (doctor, nurse/midwife, and trained community health worker) or other providers (traditional provider, family member, and pharmacist), using χ^2^ tests and multinomial regression models. Among the 4,040 adult deaths reported during 2019–2020, the major causes were HIV (17%), cancer (13%), injury (10%), cardiovascular diseases (9%), pneumonia (7%), tuberculosis (5%), and maternal causes (3%). Formal care-seeking was more likely among adults who had primary or higher level education (relative risk ratio [RRR]: 1.6, *P* < 0.001; RRR: 1.7, *P* < 0.01), were married (RRR: 1.3, *P* < 0.01), and had highest household wealth (RRR: 3.1, *P* < 0.001). Formal care-seeking was less likely among adults who were male (RRR: 0.7, *P* < 0.001), had social capital (RRR: 0.7, *P* < 0.05), or resided in the southern region (RRR: 0.4, *P* < 0.001). Information about adult causes of death is useful for formulating policy and for developing, monitoring, and evaluating programs to improve adult health in Mozambique. Care-seeking–related information helps identify barriers for seeking care from formal health providers while emphasizing the need for generating local resources and strengthening outreach health systems service delivery.

## INTRODUCTION

There is a scarcity of data to ascertain the causes of adult deaths nationally and regionally in Mozambique. A post-census mortality survey 2007–2008 reported that HIV/AIDS, malaria, circulatory diseases, tuberculosis, injury, and diarrheal diseases are among major causes of adult deaths in Mozambique.^1^ The distribution of adult causes of death varies among age groups and by sex. A systematic statistical model–based analysis to ascertain causes of death in 195 countries during 1980–2017 documented a continuing disparity in death rates by sex across different age groups amidst variability in causes of death in different age groups.^2^ Age-specific mortality patterns have changed over the years, which is documented in a study conducted in central Mozambique showing a dramatic shift to the age group 15–49 years (49%) in the post-war decade (1993–2003) compared with mortality in under-five children, which was the main contributor (58%) to all deaths during war time.^3^

In Mozambique, like many other sub-Saharan African countries, many people are born and die before being formally registered, and most demographic data are obtained using census or sample survey, including indirect estimations.^4^ Similar to most low-income countries, Mozambique does not currently have a functioning civil registration and vital statistics system that is able to produce complete and high-quality mortality data for monitoring recent trends in mortality and causes of death.^5^ Therefore, the country currently relies on national household surveys such as the Demographic and Health Surveys and the Multiple Indicator Cluster Surveys to measure mortality, but these sources are not able to generate recent and timely mortality data.^6^ The lack of the capacity to detect causes of death due to the fragility of the health system and a lack of adequate data makes it challenging to monitor mortality by cause among adults in Mozambique.^6^^,^^7^

When innovative approaches are urgently needed to support the country to effectively monitor levels and trends in mortality as well as causes of death, a cause of death may be ascertained using information obtained from bereaved relatives through verbal autopsy (VA). A cause of death may be assigned either by physician review of the VA data or following a set of predefined diagnostic criteria given in an algorithm.^8^ Also, to understand the social barriers and factors related to the deaths, a social autopsy (SA) can be implemented to identify bottlenecks in the family and community related to the lack of prompt formal care-seeking and to increase and facilitate response by the community. The integration of the VA and SA tools permits a simultaneous collection of these two types of information and the ability to correlate them for improved understanding of social, economic, and cultural factors related to specific causes of death.^9^

In this context, in January 2017, Mozambique launched the Countrywide Mortality Surveillance for Action (COMSA) to establish a national sampling registration system to monitor mortality and causes of death at the national and subnational level, including the use of VA to ascertain the causes of death. Knowledge of the causes of death helps governments and their partners to allocate resources and make decisions to identify disease prevention priorities. This article analyzes data collected through verbal and social autopsy of adult deaths under the COMSA project and provides countrywide (national and regional level) cause-specific information of adult deaths and adult care-seeking that is useful for formulating policy and developing programs to improve adult health in Mozambique.

The objectives of this study are to 1) identify the biological causes of adult deaths in Mozambique, 2) identify the social causes of adult deaths, and 3) examine care-seeking during illnesses among deceased adults in Mozambique.

## MATERIALS AND METHODS

Our verbal and social autopsy of adult deaths includes the analysis of data collected through a structured questionnaire from a nationally representative sample of 700 clusters randomly selected within each of Mozambique’s 11 provinces.

In this paper, we analyzed deaths of persons aged 18 years (as considered adult age in Mozambique) and above, stratified into 18–49 years and 50+ years, for a total of 4,040 deaths reported during years 2019–2020. We used the InSilicoVA method from the openVA R package, an improved version of the InterVA method, to classify biological causes of adult deaths.^10^

In our analysis, we grouped individual causes into broader groups. These include cancer, cardiovascular diseases, HIV, injury, maternal causes, pneumonia, tuberculosis, other causes, and other infections. Cardiovascular diseases included ischemic heart disease and stroke. Other causes of death included other and unspecified noncommunicable diseases, acute abdomen disease, acute cardiac disease, asthma, chronic obstructive pulmonary disease, chronic respiratory illness, diabetes mellitus, epilepsy, liver cirrhosis, renal failure, sickle cell with crisis, other and unspecified cardiac disease, and severe malnutrition. Other infections included dengue fever, hemorrhagic fever (non-dengue), measles, meningitis and encephalitis, other and unspecified infections, pertussis, sepsis (non-obstetric), tetanus, malaria, and diarrhea.

In this analysis, we have examined social autonomy and social capital as independent variables if they played a role in care-seeking of the deceased. Social autonomy has been defined as being an active participant of community groups including vocational training group; savings group or microcredit program; community cooperative, such as an agricultural cooperative; political group; religious group; sports club; youth/student club; women’s group; and other groups. Social capital is characterized as having people in the community working together on community issues (education/schools, health services/clinics, paid job opportunities, credit/finance, roads, public transportation, water distribution, sanitation services, agriculture, justice/conflict resolution, security/police services, mosque/church/temple, and other issues) that affect the entire or part of the community.

Principal component analysis was conducted to create a composite measure of wealth index from 20 variables concerning household’s ownership of assets. It was then divided into tertiles to categorize the lowest, middle, and highest levels in terms of household wealth.^11^

We used the Pathway to Survival model to analyze the steps of care-seeking and possible breakdowns in continuity of care that may have contributed to causes of adult deaths.^12^ Responses related to care-seeking were categorized as no care-seeking, care sought at home, and care sought outside home. Seeking care outside home included care sought from a formal provider (doctor, nurse/midwife, and trained community health worker), care sought from an informal provider (traditional providers, family members, and pharmacists), and care sought from formal or informal providers.

In addition to Pathway to Survival analysis, we have further explored socio-demographic factors that are associated with care-seeking from formal and informal providers. We have conducted multinomial regression analysis (informal, informal or formal, and formal being the outcome variables) and other socio-demographic characteristics of deceased as independent variables. A description of independent variables that were used to characterize the deceased is provided in Table 1.

**Table 1:** Description of independent variables

Characteristics (variable name)		
Age (age in years 2)	Age of deceased	Categorical (0 = 18–49, 1 = 50+)
Gender (q1203)	Sex of deceased	Categorical (0 = female, 1 = male)
Education (a4004)	Highest level of education reported	Categorical (0 = no education, 1 = primary, 2 = secondary and higher)
Marital status (a4002)	Marital status of deceased	Categorical (0 = single/divorced/separated/widowed, 1 = married/life partner)
Employment (a4007)	Employment status of deceased	Categorical (0 = unemployed, 1 = employed)
Household wealth (wlthindx1)	Twenty household possession variables used to create household wealth using principal component analysis	Categorical (0 = lowest, 1 = middle, 2 = highest)
Social autonomy (socautonomy)	Active participant of nine community groups	Categorical (0 = no, 1 = yes)
Social capital (soccapital)	People in the community worked together on community issues that affect entire or part of the community	Categorical (0 = no, 1 = yes)
Residence (residence)	Deceased lived in rural or urban area	Categorical (0 = rural 2 = urban)
Region (region)	Deceased lived in the region of Mozambique	Categorical (0 = north, 1 = central, 2 = south)

Overall, in this paper we report findings that are obtained from descriptive data analysis. To ascertain the relationship between two categorical variables, we conducted χ^2^ tests. The *P* value for significant statistical association was determined at the 0.05, 0.01, and 0.001 levels.

## RESULTS

### Socio-demographic characteristics of deceased adults.

Of the 4,040 total adult deaths, about half (51%) were males. The proportion of male deaths was higher in the age group 18–49 years (52.66%). The older age group was equally distributed between males and females. Over 45% of deceased adults did not have any education, 44% had primary level education, and the proportion was significantly higher in the age group 18–49 years compared with the age group 50+ years (51.0% versus 39.11%). About 56% of deceased adults were married, significantly higher in age group 18–49 years (61.95%) compared with the 50+ years age group (51.97%). Nearly 28% of all adults were used, although the proportion was significantly lower in the 50+ years age group (25.23%) compared with their counterparts (30.99%). The household wealth of deceased adults was divided into tertiles.

Nearly half of deceased adults were reported to have social autonomy, and about 78% possessed social capital. Overall, 73% lived in rural areas and 27% in urban areas. The proportion of deceased adults in the 50+ years age group was significantly higher in rural areas compared with that in the 18–49 years age group (73.48% versus 71.94%). The majority of adults came from the central region (43.60%), and the proportion from this region is significantly higher in the 18–49 years age group compared with the 50+ years age group (50.21% versus 38.84%) (Table 2).

**Table 2:** Socio-demographic characteristics of the adults who died of different causes, Mozambique 2019–2020

Characteristics	Total adult deaths (*N* = 4,040), %	Deaths in age group 18–49 (*n* = 1,688), %	Deaths in age group 50+ (*n* = 2,352), %	*P* value
Sex				
Female	49.00	47.34	50.20	< 0.05
Male	51.00	52.66	49.80	
Education				
No education	45.53	29.43	57.14	< 0.001
Primary	44.07	50.96	39.11	
Secondary and higher	10.39	19.60	3.75	
Marital status				
Single/divorced/separated/widowed	43.85	38.05	48.03	< 0.001
Married/life partner	56.15	61.95	51.97	
Employment				
Unemployed	72.36	69.01	74.77	< 0.001
Employed	27.64	30.99	25.23	
Household wealth				
Lowest	36.16	35.90	36.34	0.257
Middle	33.03	33.14	32.96	
Highest	30.81	30.96	30.70	
Social autonomy*				
No	50.08	51.49	49.06	0.478
Yes	49.92	48.51	50.94	
Social capital†				
No	22.51	25.12	20.62	0.066
Yes	77.49	74.88	79.38	
Residence				
Rural	72.84	71.94	73.48	< 0.05
Urban	27.16	28.06	2652	
Region				
North	24.19	26.38	22.61	< 0.001
Central	43.60	50.21	38.84	
South	32.21	23.41	38.56	

* Active participant of community groups.

† People in the community worked together on community issues that affect entire or part of the community.

### Causes of adult deaths by age.

**Figure 1.**
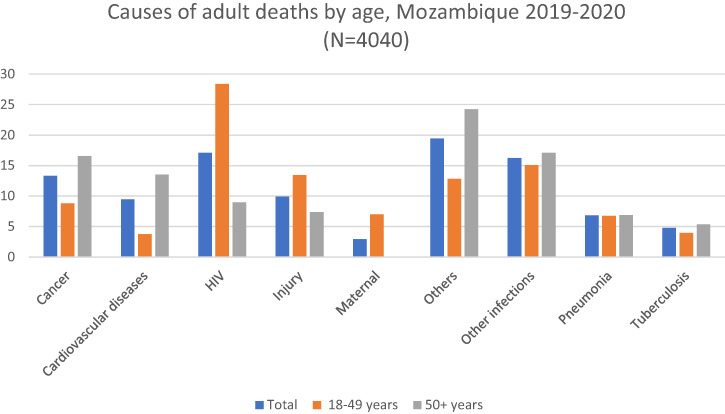
Distribution of causes of adult deaths by age, Mozambique 2019–2020. Cardiovascular diseases include ischemic heart disease and stroke. Other causes include acute abdomen, acute cardiac disease, asthma, chronic obstructive pulmonary disease, chronic respiratory illness, diabetes mellitus, epilepsy, liver cirrhosis, renal failure, sickle cell with crisis, other and unspecified cardiac disease, other and unspecified noncommunicable disease, severe malnutrition. Other infections include Dengue fever; hemorrhagic fever (non-dengue); measles; meningitis and encephalitis; other and unspecified infections; pertussis; sepsis (non-obstetric); and tetanus, malaria, and diarrhea.

The major causes of adult deaths were HIV (17.09%), cancer (13.3%), injury (9.92%), cardiovascular diseases (9.45%), pneumonia (6.84%), tuberculosis (4.78%), maternal causes (2.92%), other infections (16.25%), and other causes (19.45%). HIV causes more deaths at age 18–49 (28.36%) than at age 50+ (8.96%). Likewise, injury was more common at age 18–49 (13.43%) than at age 50+ (7.39%) On the other hand, deaths due to cardiovascular diseases were more common at age 50+ (13.52%) than at age 18–49 (3.79%), as were deaths due to cancer (16.56% versus 8.78%), tuberculosis (5.37% versus 3.97%), other infections (17.1% versus 15.07%), and other causes (24.21% versus 12.84%). Among those 18–49 years old, about 6.98% of deaths were maternal deaths; this category does not apply at age 50+ years (Figure 1).

### Causes of adult deaths by sex and place of residence.

**Figure 2.**
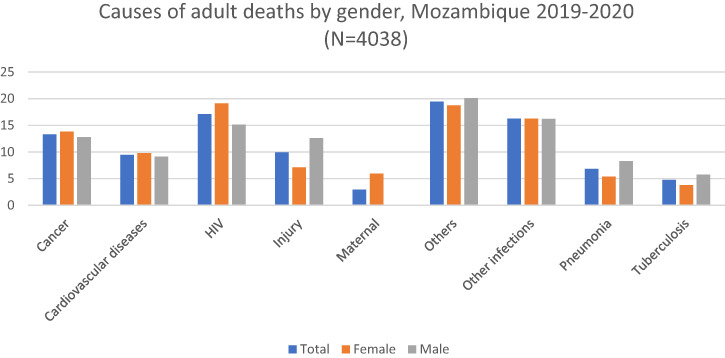
Causes of adult deaths by sex, Mozambique 2019–2020. Cardiovascular diseases include ischemic heart disease and stroke. Other causes include acute abdomen, acute cardiac disease, asthma, chronic obstructive pulmonary disease, chronic respiratory illness, diabetes mellitus, epilepsy, liver cirrhosis, renal failure, sickle cell with crisis, other and unspecified cardiac disease, other and unspecified noncommunicable disease, and severe malnutrition. Other infections include Dengue fever; hemorrhagic fever (non-dengue); measles; meningitis and encephalitis; other and unspecified infections; pertussis; sepsis (non-obstetric); and tetanus, malaria, and diarrhea.

When stratified by sex, the proportions of deaths due to cancer were significantly higher among women as opposed to men (13.81% versus 12.82%, *P* < 0.001), with similar patterns for HIV (19.12% versus 15.15%, *P* < 0.001) and cardiovascular diseases (9.81% versus 9.11%, *P* < 0.001). On the other hand, more male deaths were attributed to injury (12.6% versus 7.13%), pneumonia (8.27% versus 5.36%), tuberculosis (5.74% versus 3.79%), and other causes (20.09% versus 18.74%). The proportion of maternal deaths among women was 5.97% (Figure 2).

**Figure 3.**
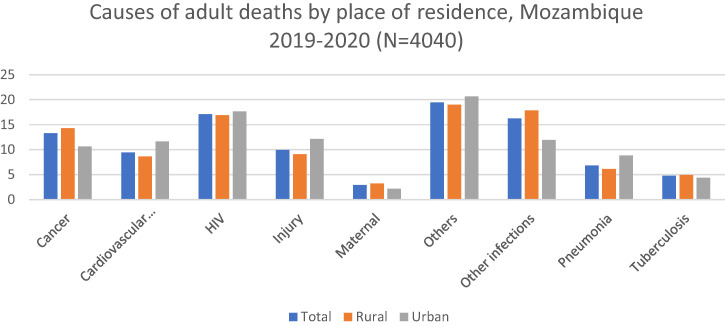
Causes of adult deaths by place of residence, Mozambique 2019–2020. Cardiovascular diseases include ischemic heart disease and stroke. Other causes include acute abdomen, acute cardiac disease, asthma, chronic obstructive pulmonary disease, chronic respiratory illness, diabetes mellitus, epilepsy, liver cirrhosis, renal failure, sickle cell with crisis, other and unspecified cardiac disease, other and unspecified noncommunicable disease, and severe malnutrition. Other infections include Dengue fever; hemorrhagic fever (non-dengue); measles; meningitis and encephalitis; other and unspecified infections; pertussis; sepsis (non-obstetric); and tetanus, malaria, and diarrhea.

Comparing adults of rural residence with those of urban residence, rural residents had higher proportions of deaths due to cancer (14.3% versus 10.62%), maternal causes (3.2% versus 2.17%), and other infections (17.85% versus 11.95%). Residents of urban areas had higher proportions of deaths due to injury (12.15% versus 9.09%), cardiovascular diseases (11.64% versus 8.63%), and other causes (20.62% versus 19.01%) (Figure 3).

### Causes of adult deaths by region and province.

**Figure 4.**
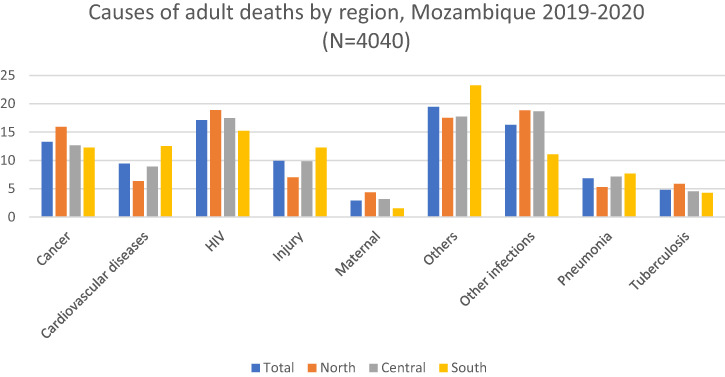
Causes of adult deaths by region, Mozambique 2019–2020. Cardiovascular diseases include ischemic heart disease and stroke. Other causes include acute abdomen, acute cardiac disease, asthma, chronic obstructive pulmonary disease, chronic respiratory illness, diabetes mellitus, epilepsy, liver cirrhosis, renal failure, sickle cell with crisis, other and unspecified cardiac disease, other and unspecified noncommunicable disease, and severe malnutrition. Other infections include Dengue fever; hemorrhagic fever (non-dengue); measles; meningitis and encephalitis; other and unspecified infections; pertussis; sepsis (non-obstetric); and tetanus, malaria, and diarrhea.

In the northern region, the proportion of deaths due to cancer (15.91%), HIV (18.9%), maternal causes (4.36%), and tuberculosis (5.85%) were higher than in other regions. The southern region included the highest proportions of deaths due to cardiovascular diseases (12.52%), injury (12.24%), pneumonia (7.65%), and other causes (23.23%) compared with other regions (Figure 4).

**Figure 5.**
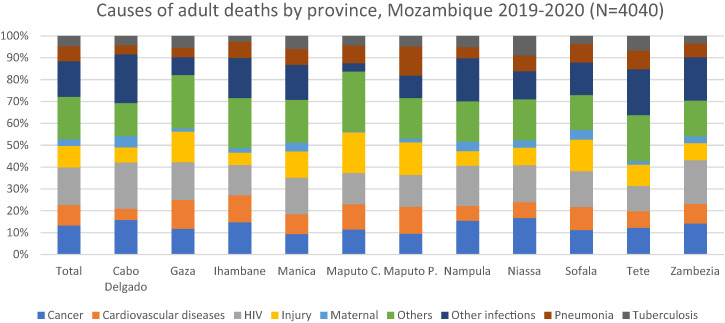
Distribution of causes of adult deaths by province, Mozambique 2019–2020 (*N* = 4,040). Cardiovascular diseases include ischemic heart disease and stroke. Other causes include acute abdomen, acute cardiac disease, asthma, chronic obstructive pulmonary disease, chronic respiratory illness, diabetes mellitus, epilepsy, liver cirrhosis, renal failure, sickle cell with crisis, other and unspecified cardiac disease, other and unspecified noncommunicable disease, and severe malnutrition. Other infections include Dengue fever; hemorrhagic fever (non-dengue); measles; meningitis and encephalitis; other and unspecified infections; pertussis; sepsis (non-obstetric); and tetanus, malaria, and diarrhea.

Figure 5 shows the distribution of causes of all deaths by 11 provinces of Mozambique compared with the national average. Proportions of cancer deaths were higher in Cabo Delgado (15.8%), Inhambane (14.81%), Zambezia (14.25%), Niassa (16.77%), and Nampula (15.53%). The proportions of deaths due to HIV were higher in Zambezia (20.08%), Nampula (18.44%), Gaza (17.26%), and Cabo Delgado (21.17%). The proportions of injury deaths were higher in Gaza (13.93%), Manica (12.08%), Maputo city (18.57%), Maputo province (14.87%), and Sofala (14.43%). The proportions of maternal deaths were higher in Cabo Delgado (5.16%), Manica (4.0%), Nampula (4.4%), Niassa (3.29%), Sofala (4.42%), and Zambezia (3.07%). The proportions of deaths associated with cardiovascular diseases were higher in Gaza (13.25%), Inhambane (12.35%), Maputo city (11.52%), Maputo province (12.28%), and Sofala (10.5%). The proportions of tuberculosis deaths were higher in Gaza (5.44%), Manica (6.01%), Maputo province (4.88%), Nampula (5.23%), Niassa (8.97%), and Tete (6.71%). The proportions of pneumonia deaths were higher in Ihambane (7.45%), Manica (7.25%), Maputo city (8.15%), Maputo province (13.23%), Niassa (7.21%), Sofala (8.43%), and Tete (8.48%), compared with the national average (Figure 5).

### Causes of adult deaths by place of deaths.

**Figure 6.**
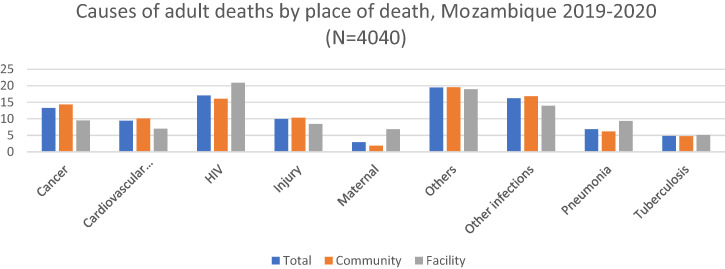
Causes of adult deaths by place of deaths, Mozambique 2019–2020. Cardiovascular diseases include ischemic heart disease and stroke. Other causes include acute abdomen, acute cardiac disease, asthma, chronic obstructive pulmonary disease, chronic respiratory illness, diabetes mellitus, epilepsy, liver cirrhosis, renal failure, sickle cell with crisis, other and unspecified cardiac disease, other and unspecified noncommunicable disease, and severe malnutrition. Other infections include Dengue fever; hemorrhagic fever (non-dengue); measles; meningitis and encephalitis; other and unspecified infections; pertussis; sepsis (non-obstetric); and tetanus, malaria, and diarrhea.

Of all deaths, 79.14% took place in the community (at home, en route to hospital, or other places in the community); the rest (20.86%) occurred in a health facility. Data showed variability in place of deaths (facility versus Community) when analyzed by causes of death. Higher proportions of deaths due to HIV, pneumonia, and tuberculosis took place in the health facilities compared with the community (20.89% versus 16.08%, 9.36% versus 6.18%, and 5.11% versus 4.7%, respectively). On the other hand, higher proportions of deaths due to cancer, cardiovascular diseases, injury, other infections, and other causes occurred in the community compared with the facility (14.3% versus 9.51%, 10.09% versus 7.01%, 10.32% versus 8.39%, 16.85% versus 13.96%, and 19.59% versus 18.93%, respectively) (Figure 6).

### Pathway to survival indicators/components.

**Figure 7.**
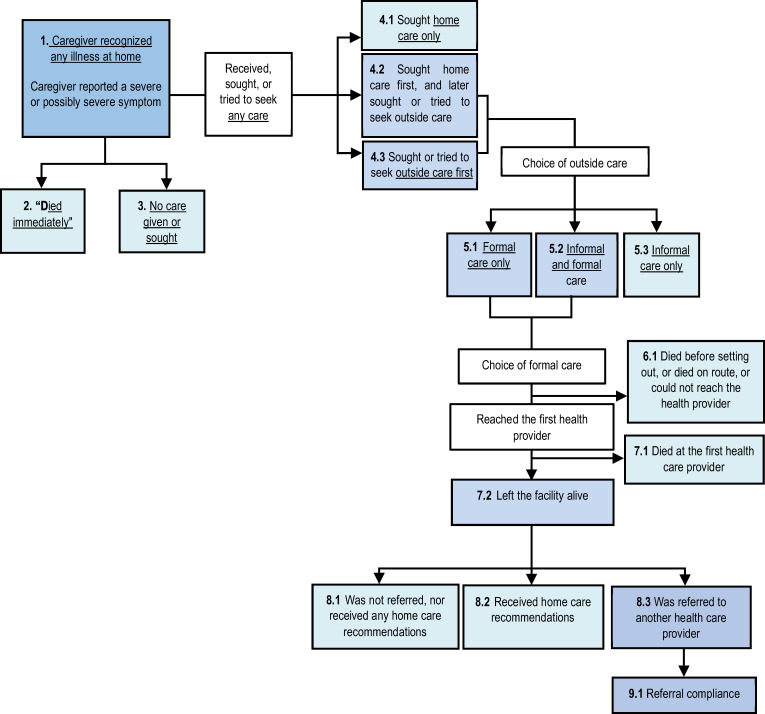
“Pathway to Survival” components and indicators.

Figure 7 illustrates the steps and possible breakdowns in the Pathway to Survival that may have contributed to death. Table 3 shows the distribution of these indicators by age group. When the deceased adults or their caregivers first noticed that they (deceased adults) were ill, healthcare was obtained or sought outside the home as a first action for most deaths (71.62%). The distribution is similar across age groups 18–49 years and 50+ years. For nearly one-fifth of adult deaths, the caregiver did not seek care after the illness was noticed (19.75%), and the proportion is higher for adults aged 18–49 years (22.04%) compared with adults aged 50+ years (18.10%). About 4% of deceased adults received only home care. Receiving home care was much higher among deceased adults aged 50+ years (5.30%) as opposed to adults aged 18–49 years (2.27%). For those who sought or tried to seek any outside care, the majority sought formal care only (66.90%); however, the proportion of formal care-seeking was higher in the younger adult group (18–49 years) (72.85%) compared with older adult group (62.66%). Contrastingly, informal care-seeking was higher among adults aged 50+ years (9.64%) compared with adults aged 18–49 years (3.93%). A combination of informal and formal care was sought more among deceased older adults (50+ years) compared with deceased younger adults (18–49 years) (27.29% versus 22.59%).

**Table 3:** Pathway to Survival indicators/components of adult deaths, Mozambique 2019–2020

Pathway to survival components	Total adult deaths (*N* = 4,040), %	Adult deaths, 18–49 years (*n* = 1,688), %	Adult deaths, 50+ years (*n* = 2,352), %
Care-seeking patterns			
3. No care given or sought for deceased	19.75	22.04	18.10
4.1 Deceased received home care only	4.03	2.27	5.30
4.2 Deceased sought or tried to seek outside care as first action	71.62	72.11	71.27
4.3 Deceased sought outside care as second action	4.51	3.44	5.28
Don’t know	0.09	0.14	0.05
Choice of outside care	(*N* = 3,076)	(*n* = 1,275)	(*n* = 1,800)
5.1 Formal care only	66.90	72.85	62.66
5.2 Informal and formal care	25.33	22.59	27.29
5.3 Informal care only	7.27	3.93	9.64
Missing	0.50	0.63	0.42
Choice of any formal care	(*N* = 2,843)	(*n* = 1,222)	(*n* = 1,621)
6.1 Died en route, or could not reach the health care provider	1.41	0.91	1.79
7.1 Reached the first health care provider and died at the facility	12.69	16.41	9.87
7.2 Reached the first health provider and left the facility alive	85.90	82.67	88.34
Decision of health provider at discharge	(*N* = 2,442)	(*n* = 1,010)	(*n* = 1,432)
8.1 Deceased was not referred, nor received any home care recommendations	34.67	31.64	36.82
8.2 Received home care recommendations	47.17	48.01	46.57
8.3 Deceased was referred to another health care provider	18.16	20.35	16.60
The caregiver followed the recommendation and went to a second or last provider	(*N* = 443)	(*n* = 206)	(*n* = 238)
9.1 Referral compliance (deceased visited health provider where he/she was referred)	85.19	88.42	82.38

Among adults who reached the first health provider, 12.7% died at the facility. The vast majority of deceased adults left the first health provider/facility alive (85.90%). Only 18.16% of adults who reached and left the first provider alive were referred to a second provider, and the rest either received home care or did not receive any home care recommendation. A majority of those who were referred complied and went to a second health provider (85.19%), and the proportion was higher among deceased adults aged 18–49 years (88.42%) compared with deceased adults aged 50+ years (82.38%) (Table 3).

### Care-seeking among deceased adults.

Table 4 shows the proportional distribution of formal care-seeking by characteristics of deceased adults. We have analyzed the data between age groups 18–49 years and 50+ years. The distribution of care-seeking from formal providers by sex was similar. However, a higher proportion of female adults (50.41%) in age group 18–49 years and a higher proportion of male adults (50.75%) in age group 50+ years sought care from formal providers. Of 2,056 deceased adults who sought care from formal providers, 40% had no formal education, 46.59% had primary level education, and 13.43% had secondary or higher education. The proportions of primary or secondary or higher were higher among the adults in age group 18–49 years compared with older adults (51.54% versus 42.50% and 22.99% versus 5.51%). Nearly 60% of the deceased adults who were married or had a life partner had sought care from a formal provider, and the proportion was higher among those aged 18–49 years (63.67%) and lower among those aged 50+ years (54.07%). Less than one-third of the deceased adults who were employed and nearly two-thirds of the deceased adults who resided in rural areas sought care from formal providers. Over half of the deceased adults having social autonomy and nearly three-fourths having social capital sought care from formal providers. The majority of the deceased adults that came from central region sought care from formal providers, and the proportion was higher in the 18–49 age group (48.27%) than in the 50+ age group (41.55%).

**Table 4:** Care seeking from formal providers for illnesses among deceased adults, Mozambique 2019–2020

Characteristics	Sought care from formal providers, %		
	Total (*N* = 2,056)	19–49 years (*n* = 929)	50+ years (*n* = 1,127)
Sex*			
Female	49.78	50.41	49.25
Male	50.22	49.59	50.75
Education			
No education	39.98	25.46	51.99
Primary	46.59	51.54	42.50
Secondary and higher	13.43	22.99	5.51
Marital status			
Single/divorced/separated/widowed	41.58	36.33	45.93
Married/life partner	58.42	63.67	54.07
Employment			
Unemployed	72.60	69.21	75.41
Employed	27.40	30.79	24.59
Household wealth			
Lowest	31.53	29.92	32.87
Middle	29.94	32.71	27.65
Highest	38.52	37.37	39.48
Social autonomy†			
No	50.62	50.03	51.11
Yes	49.38	49.97	48.89
Social capital‡			
No	27.58	29.40	26.09
Yes	72.42	70.60	73.91
Residence			
Rural	66.37	64.86	67.62
Urban	33.63	35.14	32.38
Region			
North	26.25	28.20	24.63
Central	44.60	48.27	41.55
South	29.16	23.53	33.82

* Two intersex individuals were excluded from the analysis.

† Active participant of community groups.

‡ People in the community worked together on community issues that affect entire or part of the community.

### Factors associated with care-seeking among deceased adults.

We conducted multivariate analysis to ascertain factors associated with seeking care among adults before their death. We compared the factors associated with seeking care from informal providers or from informal and formal providers combined (Table 5). Also, we compared care sought from formal providers as opposed to informal providers in relation to these factors (Table 6).

**Table 5:** Association between care seeking from health care providers and other characteristics among deceased adults, Mozambique 2019–2020 (informal vs. informal or formal)

Characteristics	RRR	Robust	Z	*P* value	95% CI
		SE			
Age at death					
19–49 years (reference)	–	–	–	–	–
50+ years	1.267	0.148	2.02	0.044	1.007–1.593
Sex*					
Female (reference)	–	–	–	–	–
Male	0.687	0.083	−3.10	0.002	0.541–0.871
Education					
No education (reference)	–	–	–	–	–
Primary	1.426	1.773	2.85	0.004	1.118–1.820
Secondary and higher	1.134	0.259	0.55	0.579	0.726–1.775
Marital status					
Single/divorced/separated/widowed (reference)	–	–	–	–	–
Married/life partner	1.247	0.145	1.90	0.058	0.992–1.567
Employment					
Unemployed (reference)	–	–	–	–	–
Employed	1.106	0.135	0.82	0.412	0.870–1.405
Household wealth					
Lowest (reference)	–	–	–	–	–
Middle	1.236	0.158	1.65	0.098	0.961–1.588
Highest	2.363	0.417	4.87	0.000	1.672–3.341
Social autonomy†					
No (reference)	–	–	–	–	–
Yes	1.142	0.127	1.19	0.233	0.918–1.421
Social capital‡					
No (reference)	–	–	–	–	–
Yes	1.2222	0.187	1.31	0.190	0.905–1.650
Residence					
Rural (reference)	–	–	–	–	–
Urban	0.776	0.120	−1.63	0.103	0.573–1.052
Region					
North (reference)	–	–	–	–	–
Central	0.944	0.140	−0.39	0.698	0.707–1.262
South	0.592	0.094	−3.31	0.001	0.434–0.807
_cons§	0.387	0.080	−4.60	0.000	0.259–0.580

RRR = relative risk ratio; SE = standard error.

* Two intersex individuals were excluded from the analysis.

† Active participant of community groups.

‡ People in the community worked together on community issues that affect entire or part of the community.

§ _cons estimates baseline relative risk for each outcome.

**Table 6:** Association between care seeking from health care providers and other characteristics among deceased adults, Mozambique 2019–2020 (informal vs. formal)

Characteristics	RRR	Robust	Z	*P* value	95% CI
		SE			
Age at death					
19–49 years (reference)	–	–	–	–	–
50+ years	1.029	0.101	0.29	0.773	0.848–1.248
Sex*					
Female (reference)	–	–	–	–	–
Male	0.679	0.067	−3.92	0.000	0.559–0.824
Education					
No education (reference)	–	–	–	–	–
Primary	1.569	0.162	4.35	0.000	1.281–1.922
Secondary and higher	1.702	0.316	2.86	0.004	1.183–2.450
Marital status					
Single/divorced/separated/widowed (Reference)	–	–	–	–	–
Married/life partner	1.299	0.125	2.71	0.007	1.075–1.569
Employment					
Unemployed (reference)	–	–	–	–	–
Employed	0.971	0.100	−0.29	0.775	0.794–1.188
Household wealth					
Lowest (reference)	–	–	–	–	–
Middle	1.216	0.130	1.82	0.069	0.985–1.500
Highest	3.140	0.436	8.24	0.000	2.391–4.122
Social autonomy†					
No (reference)	–	–	–	–	–
Yes	1.046	0.096	0.49	0.624	0.874–1.251
Social capital‡					
No (reference)	–	–	–	–	–
Yes	0.674	0.077	−3.46	0.001	0.539–0.843
Residence					
Rural (reference)	–	–	–	–	–
Urban	1.227	0.145	1.73	0.083	0.974–1.547
Region					
North (reference)	–	–	–	–	–
Central	0.933	0.111	−0.58	0.563	0.739–1.179
South	0.428	0.054	−6.68	0.000	0.334–0.549
_cons§	1.627	0.268	2.95	0.003	1.178–2.248

RRR = relative risk ratio; SE = standard error.

* Two intersex individuals were excluded from the analysis.

† Active participant of community groups.

‡ People in the community worked together on community issues that affect entire or part of the community.

§ _cons estimates baseline relative risk for each outcome.

Adults in the 50+ years age group and adults who had primary level education were significantly more likely to have sought care from informal or formal providers than to have sought care from informal providers (relative risk ratio [RRR]: 1.27, *P* < 0.05; RRR: 1.43, *P* < 0.05). Compared with deceased female adults, male adults were significantly less likely to have sought care from informal or formal providers compared with informal providers alone (RRR: 0.69, *P* < 0.01). Adults from the highest tertile were more likely than those from the lowest tertile to have sought care from informal or formal providers compared with informal providers (RRR: 2.36, *P* < 0.001). Deceased adults from southern region were significantly less likely to have sought care from informal or formal providers compared with those from the northern region (RRR: 0.59, *P* < 0.05) (Table 5).

Comparing care-seeking from informal with formal providers, adults who had had primary, secondary, or higher level of education were more likely to have sought care from formal providers than to have sought care from informal providers (RRR: 1.57, *P* < 0.001; RRR: 1.70, *P* < 0.01). Compared with deceased female adults, deceased male adults were less likely to have sought care from formal providers than to have sought care from informal providers (RRR: 0.68, *P* < 0.001). Adults who were married or had had a life partner as opposed to those who were single, divorced, separated, or widowed were more likely to have sought care from formal providers as opposed to informal providers (RRR: 1.30, *P* < 0.01). Adults who had the highest household wealth were 3.14 times more likely to have sought care from formal providers than those having the lowest household wealth (RRR: 3.14, *P* < 0.001). Social capital seemed to have a negative association with care-seeking from formal providers as opposed to informal providers (RRR: 0.73, *P* < 0.05). Compared with adults from the northern region, adults from the southern region were less likely to have sought care from formal providers than to have sought care from informal providers (RRR: 0.43, *P* < 0.001) (Table 6).

## DISCUSSION

The major causes of adult deaths in Mozambique are HIV (17.09%), cancer (13.3%), injury (9.92%), cardiovascular diseases (9.45%), pneumonia (6.84%), tuberculosis (4.78%), and maternal causes (2.92%). HIV and injury cause more deaths in younger adults (18–49 years), whereas cancer, cardiovascular diseases, pneumonia, and tuberculosis cause more deaths among adults 50+ years of age. When stratified by sex, among all adult deaths, HIV, cancer, and cardiovascular diseases appear to cause more deaths in females compared with males, for whom injury, pneumonia, and tuberculosis were the main causes.

The post-census mortality survey 2007–2008, Mozambique conducted about 12 years prior to our study reported HIV/AIDS (40%), malaria (14%), circulatory diseases (7%), tuberculosis (6%), injury (6%), and diarrheal diseases (3%) as the major causes of deaths among adults age 15 and older.^1^ The post-census mortality survey also used VA but assigned cause of death through independent review of each VA questionnaire by two trained physicians. In our study, cardiovascular diseases include ischemic heart disease and stroke, whereas the earlier study reported circulatory diseases. One study conducted in northern Ethiopia identified causes of death using 723 verbal autopsy interviews of death of adults aged 15+ years (2009–2013), and the major causes of deaths were tuberculosis (15.9%), cerebrovascular diseases (7.3%), and accidental falls (3.9%).^13^ Even though the Ethiopian study used a methodology similar to ours, the context is different, having a lower prevalence of HIV.

In our study, HIV as a single cause appears to be the leading cause of death for both the residents in rural and urban areas because nearly one-fifth of the adult population die of HIV/AIDS. The similar findings appeared in the post-census mortality survey conducted about a decade ago, suggesting that HIV/AIDS has been the major killer among the adult population in the last decade regardless of place of residence. Whereas cancer (14.3%) and injury (9.1%) have been the second and third causes of adult deaths in rural areas, injury (12.2%) and cardiovascular diseases (11.6%) take those places in urban areas. The post-census mortality survey conducted a decade ago also demonstrated the circulatory diseases (9%) and accidents and external causes (7%) were the third and fourth leading causes of adult death in urban areas; however, the data suggest that the proportion of injury-related deaths doubled (7%–12.2%) in the last decade. Study findings suggested monitoring of injury-related deaths and developing and implementing injury prevention programs to avert injury-related deaths among those living in urban areas.^1^

In this study, HIV and cancer appear as the leading causes of deaths for all the provinces. While comparing with 2007–2008 post-census mortality survey, we observed no change in the trend during the last 12 years.^1^ However, our study demonstrates that the proportion of some causes of deaths are higher in some provinces compared with other provinces (Figure 5). This warrants development and strengthening of province-specific disease prevention strategies and implementation of the strategies.

We defined social capital considering responses about having people in the community working together on community issues that affect entire or part of community. Findings from adjusted analysis using multinomial regression models showed that social capital was positively associated with deceased adults’ care -eking from informal or formal providers compared with care-seeking from informal providers (RRR: 1.222), even though the association was not statistically significant. Interestingly, when we examined adult care-seeking from formal providers as opposed to informal providers, we found a negative association with social capital (RRR: 0.674). Adults’ social autonomy, as defined as active participation with community groups, appeared to have positively associated with care-seeking from formal providers as opposed to informal providers (RRR: 1.046) and care-seeking from informal or formal providers compared with informal providers (RRR: 1.142), although the associations were not statistically significant (Tables 5 and 6).

Bakeera et al.^14^ explored the role of social capital in utilization of healthcare services by children in Uganda. In this study, social capital was measured by assessing providers’ responses to questions related to civic trust, social support, reciprocity, or willingness of community to help each other out. Study findings from adjusted analysis controlling for potential confounding factors, including socio-demographic and socio-economic factors of service recipients and service providers, demonstrated that high levels of trust and medium levels of informational support (odds ratio [OR]: 2.75, 95% CI: 1.50–5.02; and OR: 1.68, 95% CI: 1.12–2.50, respectively) were positively associated with the use of a public facility compared with other treatment options (community medicine distributor, neighbor, drug shops, and others).

Our study findings suggest significant variability in care-seeking across regions. Compared with adults from the north region, adults from the southern region are less likely to have sought care from formal providers than to have sought care from informal providers (RRR: 0.428, *P* < 0.001) (Table 6). Similarly, compared with adults from the north region, adults from the southern region are less likely to have sought care from informal or formal providers than to have sought care from informal providers (RRR: 0.592, *P* < 0.01) (Table 5).

Care-seeking data from our study suggest that nearly one-fifth of the deceased did not seek any care before death, and 4% received home care. Nearly three-fourths sought care or tried to seek care outside the home, and the majority sought formal care. Care-seeking from formal providers was much higher among younger adults (18–49 years) compared with older adults (50+ years). The proportions of receiving home care and seeking informal care were much higher among older adults (50+ years) compared with their younger counterparts. The main barriers for care-seeking included physical distance of the health facility, unavailability of transportation to travel to health facility, and cost related to healthcare and transportation (data not shown).

Whereas physical distance of health center and transportation appear to be the main barriers for maternity care-seeking,^15^^,^^16^ access and utilization of available services depend on information, cost, and quality of services.^17^ These factors also play a critical role in healthcare seeking and healthcare utilization by adults. Furthermore, deep-rooted social beliefs, stigma, and family practices influence care-seeking for health, although practicalities of service availability and cost often outweigh the deeply held beliefs and practices.^18^

In this study, household wealth, a proxy indicator of respondent’s socio-economic status, appeared to be strongly associated with care-seeking during illnesses. Findings from multinomial regression analysis suggest, compared with the lowest tertile, respondents belonging to the highest tertile were more likely to have sought care from formal health providers as opposed to informal health providers (RRR: 3.14, *P* < 0.001). Similarly, compared with the lowest tertile, the highest tertiles were more likely to have sought care from informal or formal providers as opposed to informal providers (RRR: 2.363, *P* < 0.001).

The positive association between socio-economic status of an individual and healthcare seeking behavior is documented in other studies. A study conducted in one subdistrict in Bangladesh revealed that people living outside the embankment (considered to be poorer) were significantly less likely to seek care from medically trained provider compared with people living inside the embankment (OR: 0.56, 95% CI: 0.286–0.834).^19^ The positive association of women’s socio-economic status and care-seeking from trained providers during pregnancy and delivery has been documented in earlier studies.^20^^–^^23^

In our study, among those who reached and left the first provider alive, less than 20% were referred to a second provider, and the rest either received home care or did not receive any home care recommendation. Over four-fifths (83.24%) of those who were referred complied and went to a second health provider, and the proportion was higher among deceased adults aged 18–49 years (88.42%) compared with deceased adults aged 50+ years (82.38%) (Table 3). It is intriguing to note that despite the higher compliance rate, only 18.16% were referred. The lack of good physical infrastructure (roads, transports) and communication services especially in the rural areas of most low- and middle-income countries (LMICs) are barriers to referring and transporting patients in emergencies to higher-level health facilities.^24^ Quality of service delivery of a referral facility is also critical for the service provider for making a decision before referring a patient. Moreover, many LMICs lack an organized and active referral system. The Ministry of Health can demonstrate a leadership role in developing a functional referral system engaging public, private, and NGO health providers and facilities in LMICs.

Our care-seeking analysis used the Pathway to Survival model to report sequential steps, possible breakdowns and itinerary of care, and failures in the pathway to survival that may have contributed to causes of adult deaths. Also, we have explored and reported socio-demographic factors that are associated with care-seeking from different providers. However, one of the limitations of our study is that it included the care-seeking information of the deceased only. Another limitation of our study is possible recall bias in providing information by the respondents on death and care-seeking of the deceased prior to death.

## CONCLUSION

Findings of this study about adult causes of death are useful for program planning and development of priority programs within the limited resources available for provision of healthcare. The information is also important for monitoring and evaluation of ongoing disease prevention programs, including malaria, HIV, and tuberculosis. Regional- and provincial-level cause of death information is pivotal for local level program planning and implementation and for strengthening provincial- and district-level health systems, including disease prevention programs and community outreach services. Study findings related to adult care-seeking not only identify demand and supply-side barriers but also help Mozambique health systems develop and strengthen affordable outreach services. The information emphasizes the need for developing community-driven programs to generate local-level resources and facilitate transportation to overcome the barriers in seeking care during illnesses.

## Financial Disclosure

Financial support: The COMSA project is implemented through the generous support of the Bill & Melinda Gates Foundation through the Johns Hopkins University.
